# Two additive mechanisms impair the differentiation of 'substrate-selective' p38 inhibitors from classical p38 inhibitors in vitro

**DOI:** 10.1186/1752-0509-4-23

**Published:** 2010-03-15

**Authors:** Bart S Hendriks, Kelly M Seidl, Jeffrey R Chabot

**Affiliations:** 1 Systems Biology, Pfizer Research Technology Center, 620 Memorial Drive, Cambridge, MA, 02139, USA

## Abstract

**Background:**

The success of anti-TNF biologics for the treatment of rheumatoid arthritis has highlighted the importance of understanding the intracellular pathways that regulate TNF production in the quest for an orally-available small molecule inhibitor. p38 is known to strongly regulate TNF production via MK2. The failure of several p38 inhibitors in the clinic suggests the importance of other downstream pathways in normal cell function. Recent work has described a 'substrate-selective' p38 inhibitor that is able to preferentially block the activity of p38 against one substrate (MK2) versus another (ATF2). Using a combined experimental and computational approach, we have examined this mechanism in greater detail for two p38 substrates, MK2 and ATF2.

**Results:**

We found that in a dual (MK2 and ATF2) substrate assay, MK2-p38 interaction reduced the activity of p38 against ATF2. We further constructed a detailed kinetic mechanistic model of p38 phosphorylation in the presence of multiple substrates and successfully predicted the performance of classical and so-called 'substrate-selective' p38 inhibitors in the dual substrate assay. Importantly, it was found that excess MK2 results in a stoichiometric effect in which the formation of p38-MK2-inhibitor complex prevents the phosphorylation of ATF2, despite the preference of the compound for the p38-MK2 complex over the p38-ATF2 complex. MK2 and p38 protein expression levels were quantified in U937, Thp-1 and PBMCs and found that [MK2] > [p38].

**Conclusion:**

Our integrated mechanistic modeling and experimental validation provides an example of how systems biology approaches can be applied to drug discovery and provide a basis for decision-making with limited chemical matter. We find that, given our current understanding, it is unlikely that 'substrate-selective' inhibitors of p38 will work as originally intended when placed in the context of more complex cellular environments, largely due to a stoichiometric excess of MK2 relative to p38.

## Background

The precedence for tumor necrosis factor alpha (TNF) as a target has been well established by the anti-TNF biological therapeutics currently on the market [1]. While the biologic therapies available are targeted at TNF directly, TNF production can be regulated at intracellular several points as well, including transcription, translation and shedding from its membrane-anchored precursor on the cell surface, all of which have been pursued as drug targets by various companies [2]. p38 MAPK was originally identified as the target of a compound that regulated the production of multiple pro-inflammatory cytokines, including TNF. p38's regulation of TNF production is largely thought to be mediated via MK2, one of its many substrates. Active MK2 serves to stabilize TNF mRNA, thereby positively contributing to TNF production [3-5].

Dozens of small molecule p38 inhibitors have been put into the clinic for the treatment of chronic inflammatory diseases such as RA [6-9]. These compounds represent a diverse chemical space [6,7,9] and in spite of being highly selective [10], none has yet made it to the market, with many failing due to adverse events, most notably liver enzyme elevation and skin rashes [11]. It has been hypothesized that the adverse events may be mechanistically linked to p38 [12], possibly due to the disruption of the normal p38 function within the cell, beyond regulation of TNF production. Thus, drug discovery teams have sought means to provide a more selective inhibition of TNF production.

It is very attractive to attempt to selectively block TNF by targeting intracellular signaling mechanisms regulating its production. Further, it has been surmised that intervening proximal to TNF (as opposed to blocking signaling farther upstream) will help avoid unwanted effects. To this end, MK2 has been investigated as a potential target [13]. However, MK2 itself has proven to be a challenging molecule to selectively target with small molecules [14]. Consequently, attention has reverted to p38. Based on the established druggability of p38 as a target, its diverse role in cellular function and the specific role for MK2 in TNF production it has been hypothesized that specific modulation of this interaction would lead to an improved safety profile over previous p38 inhibitors. This is the basis for the development of so-called 'substrate-selective' inhibitors as described by Davidson, et al. [15]. In Davidson, *et al*. a 'substrate-selective' a p38-alpha inhibitor was described that prevented p38-alpha-dependent MK2 phosphorylation (K_I, app _= 330 nM) but did not prevent phosphorylation of another p38 substrate, ATF-2 (K_I, app _> 20 uM) [15]. The structural details of how this molecule is able to elicit differential inhibition of MK2 and ATF2 were not disclosed or not known. ATF2 was used as a representative nuclear localized transcription factor in this assay, even though it is known to be phosphorylated by other kinases, such as JNK [16]. ATF2 is a histone acteyltransferase that binds DNA in a sequence-specific manner [17]. It activates a variety of gene targets including cyclin A, cyclin D, and c-jun, which are involved in oncogenesis [18]. p38 phosphorylates ATF2 on Thr 69 and Thr 71 [19].

Compounds satisfying the 'substrate-selective' criteria can be discovered through high-throughput screening approaches. Two screens are set up: one looking for p38-alpha mediated MK-2 phosphorylation and one for p38alpha mediated ATF2 phosphorylation. Compounds are selected such that their potency in the MK2 assay >> ATF2 assay. Thus, by the construction of the screening campaign, such compounds are said to be 'substrate-selective'.

In this work, we investigate the degree to which 'substrate-selectivity' holds as these classes of compounds are tested under conditions with multiple competing substrates. Using a combination of biochemical experiments and kinetic modeling we explore the contributions of mechanism and stoichiometry in determining the feasibility of the 'substrate-selective' mechanism under more complex, multi-substrate conditions.

## Results

Previous work has defined a 'substrate-selective' p38 inhibitor as a compound that has a lower IC_50 _for one of its substrates than another, as assessed in independent assays [15]. This behavior has been demonstrated for a p38 inhibitor described in Davidson, et al (CMPD1) that exhibited a lower IC_50 _for MK2 than for ATF-2, both well-known substrates of p38 [15]. In order to verify this behavior two assays were developed on the Meso-Scale Discovery platform, one for the phosphorylated form of the transcription factor, ATF2 and one for phosphorylated form of the kinase MK2, as described in *Methods*. In each case, the degree of phosphorylation serves as a readout of the activity of p38 for its respective substrate.

We chose to evaluate the compound from the original Davidson paper, CMPD1 [15] with 2 traditional p38 inhibitors: SD-0006 [7] and BIRB 796 [20].

**Figure 1:**
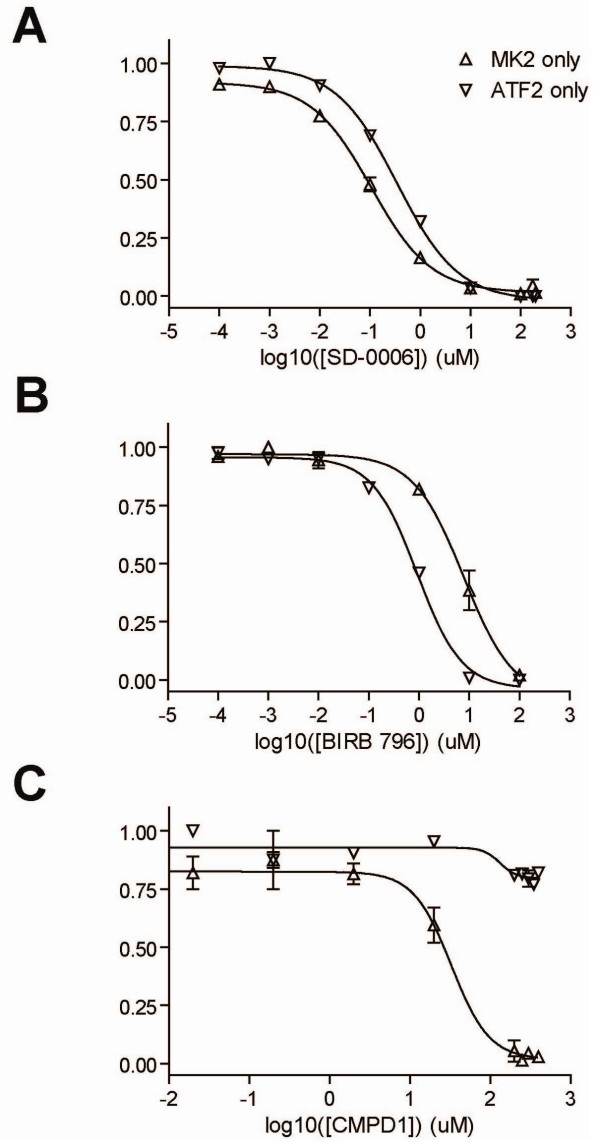
**Single substrate assay**. Phospho-MK2 (Δ) or phospho-ATF2 (▽) was measured at 30 min or 120 min, respectively in the single substrate assay. Data are shown as a fraction of the maximum signal for varying amounts of compound treatment: *A*, SD-0006, *B*, BIRB 796, *C*, CMPD1. IC_50 _values for each compound/substrate are listed in Table 1. Data is shown ± standard deviation (n = 2)

Using these assays, IC_50_'s of the 3 compounds were measured against either MK2 or ATF2, shown in Figure 1. Traditional p38 inhibitors, SD-0006 and BIRB 796, inhibited MK2 phosphorylation and ATF2 phosphorylation in a dose-dependent manner with IC_50_s within 10-fold of each other (Figure 1, Table 1). By contrast, the phospho-ATF2 dose-response curve for the CMPD1 are significantly right-shifted relative to the phospho-MK2 dose-response curve. Similar results were obtained for an in-house substrate-selective compound (data not shown).

**Table 1:** Single Substrate Assay: Compound IC_50 _Values (uM)

*Compound*	*phospho-MK2*	*phospho-ATF2*	substrate selectivity*
SD-0006	0.11 ± 1.2	0.35 ± 1.1	3.3
BIRB 796	7.75 ± 1.3	0.92 ± 1.1	0.12
CMPD1	23.4 ± 1.9	>400**	>17

IC_50_'s are shown ± standard error.

*Substrate Selectivity is defined as a relative measure between inhibiting phospho-ATF2 and phospho-MK2 = (ATF2 IC_50_)/(MK2 IC_50_). This quantity is distinct and separable from a compound's potency.

**CMPD1 is unable to completely inhibit phospho-ATF2 within its solubility limits.

### ATF2/MK2 Dual Substrate Assay

We next sought to determine how substrate-selective compounds would behave in a context where multiple competing substrates were present. To this end, we designed a dual-substrate assay in which p38 could simultaneously phosphorylate MK2 and/or ATF2. The assay conditions chosen were 0.5 nM p38, 100 nM ATF2, 10 nM MK2, 50 uM ATP. As with the single substrate assay, MK2 phosphorylation was assayed at 30 min and ATF2 phosphorylation was assayed at 120 min to ensure that each measurement was within the linear range of the assay. ATP levels were measured at the end of the assay, to confirm that it was not being depleted.

**Figure 2:**
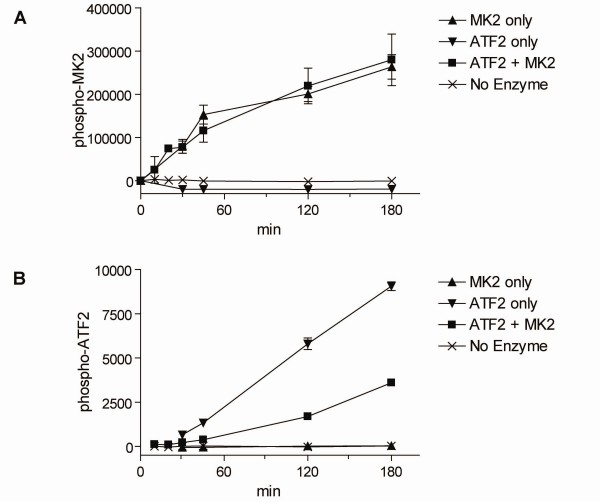
**Dual substrate assay**. *A*, the time course of MK2 phosphorylation by p38 is measured in the presence (□) or absence (Δ) of 100 nM ATF2. No MK2 (ATF2 only, ▽) and no enzyme (×) are shown as controls. *B*, the time course of ATF2 phosphorylation by p38 is measured in the presence (□) or absence (▽) of 10 nM MK2. No ATF2 (MK2 only, Δ) and no enzyme (×) are shown as controls. Data is shown ± standard deviation (n = 4).

The dual substrate assay was run in the absence of compound to examine the effect of the second substrate. In the absence of compound, the addition of ATF2 had no discernable effect on MK2 phosphorylation (Figure 2a). Conversely, we found that addition of MK2 markedly inhibited ATF2 phosphorylation, as one might expect given that MK2 has a much higher affinity for p38 than ATF2 (Figure 2b).

**Figure 3:**
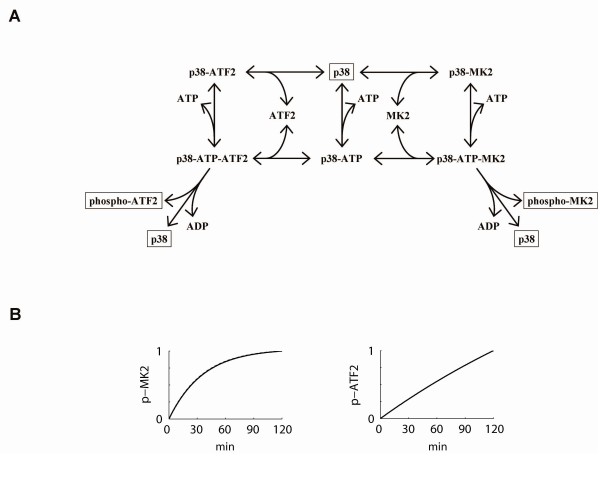
**Base kinetic model**. *A*, the base kinetic mechanism for p38 competition with two substrates in a random order bi-substrate reaction. After reversibly binding ATP and a second substrate (ATF2 or MK2) in random order, the ternary complex undergoes an irreversible phosphorylation step to create phosphorylated product, ADP and recover the active p38. Kinetic parameters are listed in Table 2. *B*, kinetic simulation of the base kinetic mechanism 0.5 nM p38, 50 uM ATP, 10 nM MK2 and 100 nM ATF2. Results are virtually indistinguishable from MK2 only condition for phospho-MK2 and from ATF2 only condition for phospho-ATF2.

In order to aid in the interpretation of the dual substrate assay, we developed a simple kinetic model of p38-mediated phosphorylation of two substrates: ATF2 and MK2 (diagrammed in Figure 3a). Since p38 has a single kinase domain and ATP binding site, we assumed that p38 can only act on a single substrate at a time. For each substrate, a random-order bi-substrate (substrate + ATP) reaction mechanism was assumed. Active p38 can reversibly bind ATP, with affinity *K*_D, ATP _and ATP binding is independent of further complex formation. Active p38 (ATP bound and unbound) can reversibly bind to ATF2 or MK2 to form complexes p38-ATF2 or p38-MK2, respectively. Each complex undergoes an irreversible catalysis step to form products phospho-ATF2 (and ADP) or phospho-MK2 (and ADP). The model equations are a set of ordinary differential equations written in terms of mass-action kinetics. Binding interactions are characterized by affinities *K*_D, ATF2 _or *K*_D, MK2_, respectively, as listed in Table 2. Catalysis rates *k*_cat, ATF2 _and *k*_cat, MK2 _are also listed in Table 2. For the case where only one substrate is present, this model reduces to the single substrate assay.

**Table 2:** Biochemical Kinetic Model Parameters

*Name*	*Description*	*Value*	*Units*	*Reference*
*K*_D, ATP_	ATP-p38 affinity	67	uM	In-house measurement
*K*_D, ATF2_	ATF2-p38 affinity	38	uM	[30]
*K*_D, MK2_	MK2-p38 affinity	20	nM	[31]
*k*_cat, ATF2_	p38-ATF2 catalytic rate	1.2	s^-1^	[30]
*k*_cat, MK2_	p38-MK2 catalytic rate	0.17 2.4	s^-1^ s^-1^	[31] In-house measurement
[p38]	p38 (active) initial condition	0.5	nM	
[ATP]	ATP initial condition	50	uM	
[ATF2]	ATF2 initial condition	100	nM	
[MK2]	MK2 initial condition	10	nM	

Using this simple competitive model, we simulated the single and dual-substrate assays (Figure 3b). The simulation results over a 120 min time scale indicate very subtle differences in ATF2 phosphorylation between the single and dual substrate assays. However, the experimental results from the dual substrate assay indicate a far more pronounced inhibition of ATF2 phosphorylation in the dual substrate assay than seen in the simulation results. Thus, this basic competitive mechanism was not quantitatively consistent with the experimental data and prompted us to examine the basic mechanism further.

**Figure 4:**
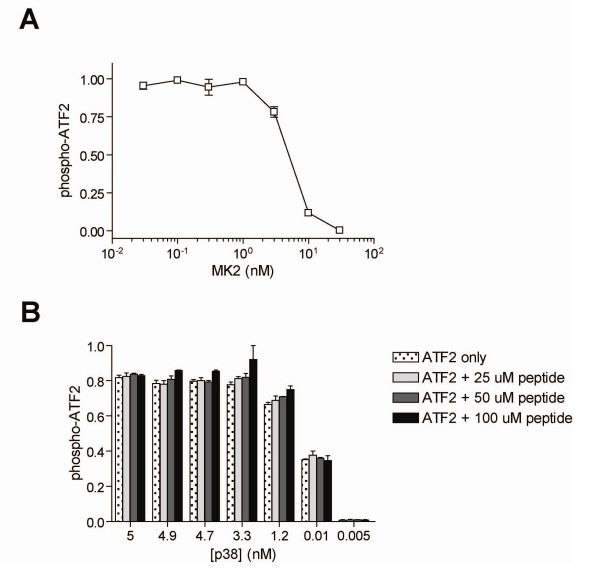
**MK2 dependence**. *A*, phosphorylation of ATF2 by p38 was measured for varying concentrations of MK2 in dual substrate assay (n = 2). *B*, phosphorylation of ATF2 by p38 was measured for in the presence of peptide 4 and for varying p38 levels (n = 4). Data is presented as the fraction of the maximum signal. Data is shown ± standard deviation.

We next experimentally measured the effect of MK2 levels on the degree of ATF2 phosphorylation for a fixed concentration of p38. This demonstrated that the inhibition of ATF2 phosphorylation by MK2 was dose-dependent. (Figure 4a). Secondly, we questioned whether the inhibition effect was due to MK2 specifically, or simply required any second p38 substrate. For this we chose to use another known p38 substrate, 'peptide 4' [21]. In our assays, the true Km of this peptide was determined to be roughly 40 uM (data not shown). The inhibition of ATF2 phosphorylation was measured in the presence of peptide 4 at 0, 25, 50 and 100 uM, and shown to have no effect on phospho-ATF2, independent of p38 levels used (Figure 4b).

**Figure 5:**
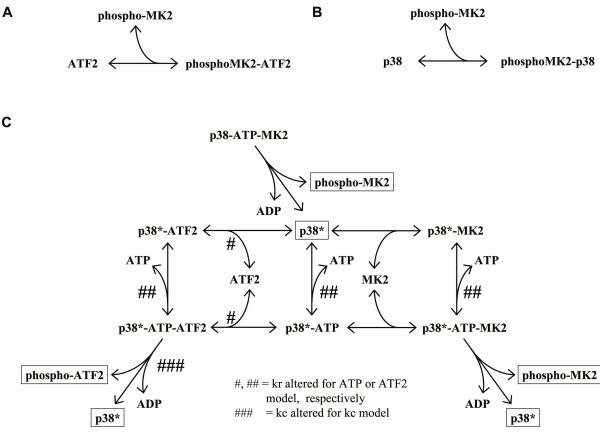
**Alternate kinetic mechanisms**. *A*, substrate inhibition (mechanism #1 in main text): MK2 and ATF2 are allowed to rebind p38 after phosphorylation. *B*, phospho-MK2 binds ATF2 (mechanism #2 in main text): phosphorylated MK2 is allowed to bind unphosphorylated ATF2 and prevent its interaction with p38. *C*, altered p38 (mechanisms #3-5 in main text): after phosphorylating MK2, p38 is left in an altered state such that it has either an altered affinity for ATP (mechanism #3 in main text; indicated by #), an altered affinity for ATF2 (mechanism #4 in main text; indicated by ##) or an altered catalytic rate for ATF2 (mechanism #5 in main text; indicated by ###).

**Figure 6:**
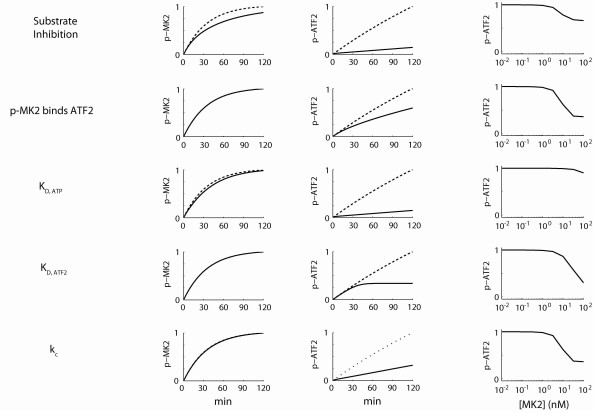
**Alternate mechanism simulations**. *A*, time courses of phospho-MK2 (1^st ^column) or phospho-ATF2 (2^nd ^column) were simulated for the kinetic mechanisms detailed in Figure 5. Time courses were simulated for the single substrate (10 nM or 100 nM ATF2, dashed line) or the dual substrate assay (solid line, 10 nM and 100 nM ATF2). *B*, the dose-dependence phospho-ATF2 was simulated as a function of MK2 for each kinetic mechanism.

In order to explain the MK2-induced inhibition of ATF2 phosphorylation seen in the experimental data, we hypothesized five alternate mechanisms: [1] phospho-MK2 was inhibiting p38 via substrate inhibition; [2] phospho-MK2 was binding ATF2 preventing its interaction with p38; or [3-5] p38 itself is modified after phosphorylating MK2 either by [3] altering its affinity for ATP, [4] altering its affinity for ATF2 or [5] altering its catalytically activity for ATF2. Each model was coded into the corresponding biochemical reaction scheme (diagrammed in Figure 5). For each reaction scheme the single and dual substrate assays were simulated (Figure 6, columns 1 & 2), as well as the dose-dependence on MK2 levels (Figure 6, column 3).

### Mechanism #1

In this mechanism, substrates are permitted to rebind to p38 after getting phosphorylated. While this mechanism successfully impedes ATF2 phosphorylation, it can nonetheless be ruled out because it is predicted to reduce overall MK2 phosphorylation. Further, this substrate inhibition mechanism fails to reproduce the observed dose-dependence on MK2 concentration (Figure 6). If phospho-MK2 stays associated with p38 it will affect p38's ability to further phosphorylate other MK2 molecules resulting in the decrease in overall p-MK2. Finally, this mechanism also did not show sufficient sensitivity to MK2 levels as seen in the MK2 dose-response curve. Intuitively this occurs because MK2 levels already exceed p38 level in the assay, consequently, p38 is quickly saturated by phospho-MK2.

### Mechanism #2

In this mechanism, MK2 is allowed to bind ATF2 and prevent its interaction with p38. Intuitively, this mechanism is limited by the stoichiometry of the assay, in which MK2 is at a 10-fold lower concentration than ATF2. Consequently, this mechanism can be ruled out because no effect was seen on MK2 in addition to an insufficient magnitude of effect on the MK2 dose-response curve at high MK2 concentrations.

### Mechanisms #3-5

The remaining mechanisms investigate 3 ways in which the activity of the p38 kinase might be altered following interaction with MK2. In mechanism #3, MK2 alters the affinity of p38 for ATP. In this case, phosphorylation of ATF2 is significantly inhibited. By contrast, phospho-MK2 is relatively unaffected due to its high affinity for p38. However, this mechanism shows little to no sensitivity to MK2 concentration and can consequently be ruled out as an independent mechanism.

Mechanisms #4 and #5 posit that MK2 alters the affinity for ATF2 (K_D, ATF2_) and the catalytic activity (k_cat_) of p38, respectively. Each parameter was assumed to be affected 10-fold. In both cases, these mechanisms are qualitatively consistent with the observed data. They have no discernable effect on phosphorylation of MK2, while dramatically inhibiting phosphorylation of ATF2. Further, each shows a dose-dependence with total MK2 concentration.

### Model validation

**Figure 7:**
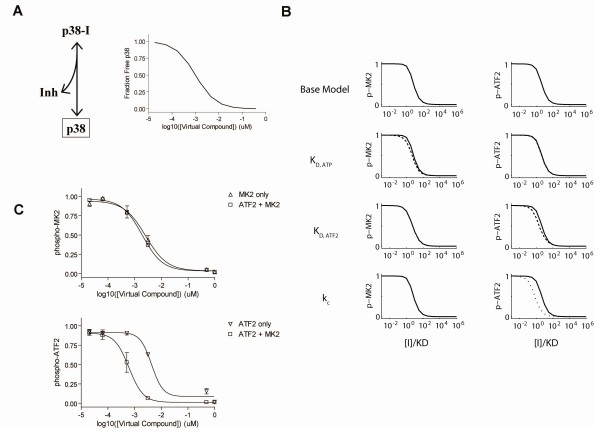
**Virtual compound experiment**. *A*, the relationship between free p38 and a 'virtual p38 inhibitor' is shown schematically. *B*, the dose-response of ATF2 phosphorylation is simulated for the virtual p38 inhibitor using multiple kinetic mechanisms. *C*, phosphorylation of MK2 (top) and ATF2 (bottom) by p38 was experimentally measured for varying concentrations of the 'virtual p38 inhibitor' under presence of 10 nM MK2 and 100 nM ATF2. The IC_50 _of the 'virtual p38 inhibitor' for phospho-MK2 and phospho-ATF2 are listed in Table 3. Data is shown ± standard deviation (n = 4).

In order to validate the model, we aimed to predict and measure the behavior of a perfectly non-substrate selective p38 inhibitor. Since we cannot be guaranteed that any of the compounds exhibit this idealized behavior we devised a 'virtual p38 compound' that could be tested experimentally. Conceptually, an ideal non-substrate-selective inhibitor of p38 would bind p38 and prevent its activity, effectively titrating out the p38. Experimentally and computationally, this could be performed by simply lowering the p38 level in concordance with a simple inhibitor-p38 binding isotherm (fraction of p38 bound by inhibitor = [Inhibitor]/([Inhibitor] + K_I_). The resulting relationship between 'virtual compound' and free p38 is shown in Figure 7a. Using the model, the virtual inhibitor is simulated. Mechanisms #4 & #5 predict a discernable left-shift in IC_50 _for the dual-substrate assay and no effect on the phospho-MK2 assay (Figure 7b). The magnitude of the shift in each case is dependent on how much the corresponding parameter is affected following MK2 interaction. The 'virtual compound' was tested in the single and dual substrate assays, shown in Figure 7c. As predicted with the kinetic model, there was no effect on the MK2 IC_50 _and a significant left-shift in the ATF2 IC_50_. Thus, the simple presence of MK2 reduces the ability of p38 to phosphorylate ATF2, even in the absence of compound. Further, this effect is qualitatively and quantitatively consistent with MK2 altering p38's affinity and/or catalytic activity for ATF2.

**Figure 8:**
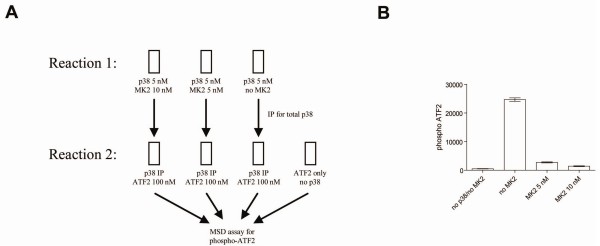
**Immunoprecipitation experiment**. *A*, Schematic of experimental design for immunoprecipitation experiment. p38 was incubated with varying amounts of MK2 as described in *Methods*. Following reaction, total p38 was immunoprecipitated and then reacted with 100 nM ATF2. *B*, ATF2 phosphorylation, following the experimental design described in A, was measured for varying MK2 concentrations.

If the p38-MK2 interaction affected p38's subsequent ability to phosphorylate ATF2, we predict that p38 should have a markedly different ability to phosphorylate ATF2 with and without pre-incubation with MK2. To test this, p38 was incubated with different amounts of MK2 for 120 min, under the same reaction conditions as in the single substrate assay. Following incubation, p38 was immunoprecipitated to remove it from MK2. The immunoprecipitates were analyzed by Western blot to demonstrate there was no MK2 contamination (data not shown). Finally, the immunoprecipitated p38 was then used in a subsequent reaction with ATF2 and phospho-ATF2 measured after 120 min of incubation time. As shown in Figure 8, pre-incubation with MK2 dramatically inhibited the ability of p38 to phosphorylate ATF2.

**Figure 9:**
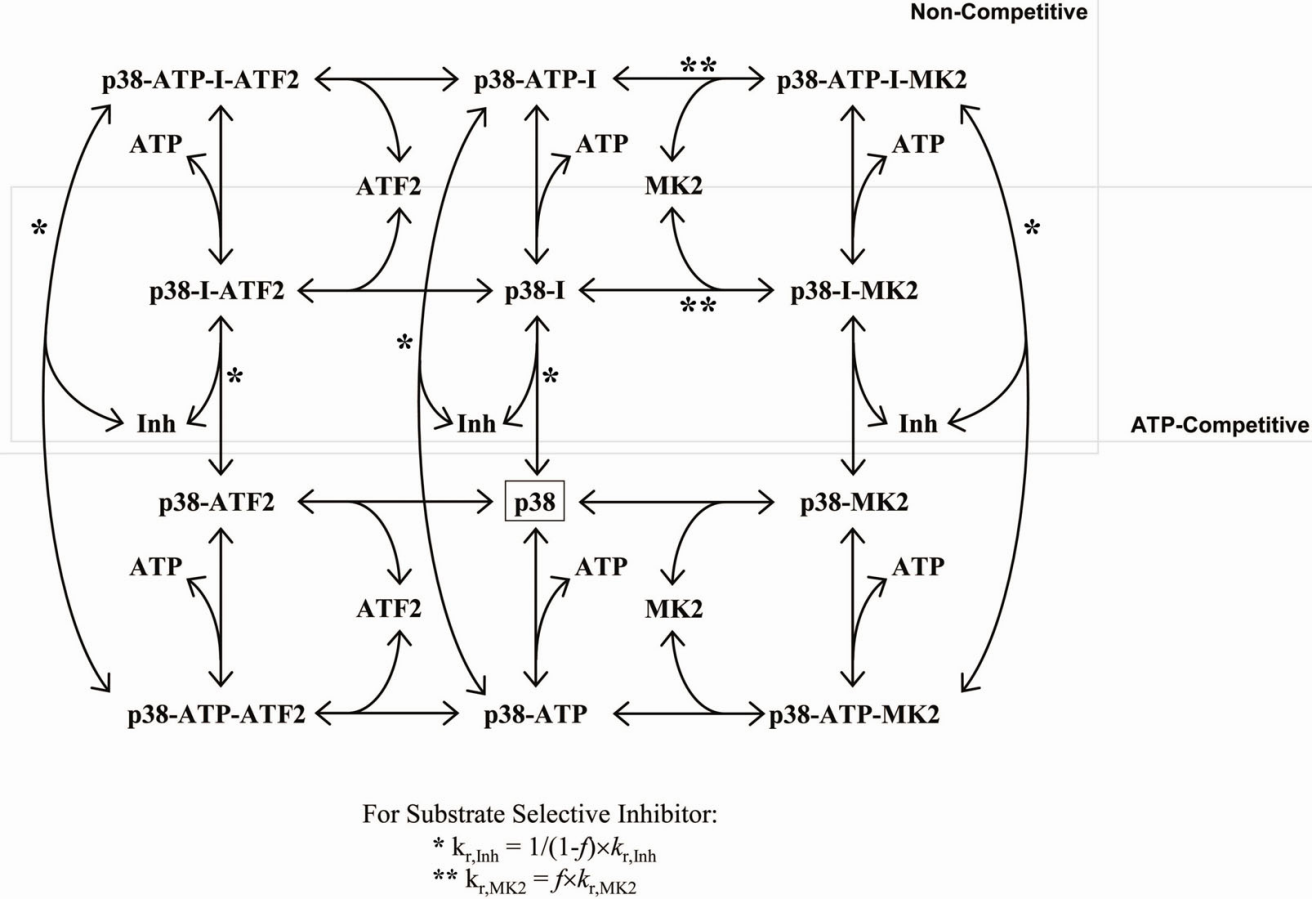
**Inhibitor kinetic mechanism**. The kinetic mechanism for a non-competitive and/or ATP-competitive p38 inhibitor is shown. Substrate-selective inhibitors are mechanistically described by altering the appropriate rate constants, indicated with * and **.

Having validated the model and developed a grasp of the potential mechanisms underlying the behavior of the dual substrate assay, we then sought to simulate the effects of both classical and substrate-selective p38 inhibitors. A classical, non-substrate-selective p38 inhibitor was simulated in a standard fashion by adding to the model an inhibitor that could interact with all forms of p38 with equal affinity, characterized by K_I_. In order to simulate the presence of a 'substrate-selective' compound we added a compound to the kinetic model that binds the p38-MK2 complex with affinity *K*_I_. The compound was allowed to bind to free p38 and p38-ATF2 with a reduced affinity *K*_*D*, I_/(1-*f*) where *f *is the selectivity (1 = perfect selectivity; 0 = no selectivity). This compound serves to stabilize the p38-MK2 complex and thus the MK2 dissociation rate constant must be multiplied by (1-*f*) to satisfy thermodynamic constraints of microscopic reversibility. For our purposes, we assumed a highly potent and selective compound with *K*_I _= 1 nM and *f *= 0.99. The details of kinetic mechanisms are cartooned in Figure 9. Both ATP-competitive and ATP non-competitive compounds were simulated.

**Figure 10:**
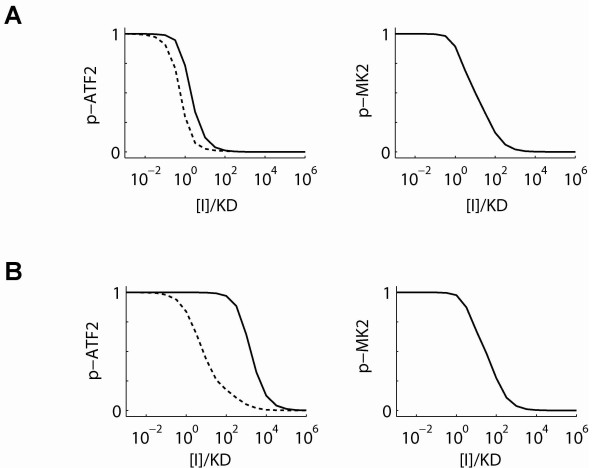
**Inhibitor simulations**. The dose-response of phospho-ATF2 and phospho-MK2 are simulated for *A*, classical p38 inhibitors and *B*, substrate-selective p38 inhibitors. Solid lines indicate results from single substrate assay and dashed lines indicate results for dual substrate assay. Association rate constants of inhibitors are assumed to be diffusion-limited and inhibitor concentrations are normalized by their affinities (KD).

Dose-response curves were simulated for classical and substrate-selective inhibitors, predicting the effects on phospho-ATF2 and phospho-MK2 for both the single and dual substrate assays (Figure 10). Inhibitor dose-response curves for the phospho-ATF2 and phospho-MK2 measurements were generated at the 120 and 30 min time points, respectively. The model simulations predict that as one moves from the single substrate assay to the dual substrate assay, there will be a left-shift in phospho-ATF2 IC_50 _for the classical p38 inhibitors and an even greater left-shift for the substrate-selective inhibitors. Meanwhile, the simulations predicted no change in the phospho-MK2 IC_50 _between the single and dual substrate assay for either inhibitor. Simulation results are shown in Figure 10. Both ATP-competitive and ATP non-competitive compounds had qualitatively indistinguishable results.

### Compound Evaluation

**Figure 11:**
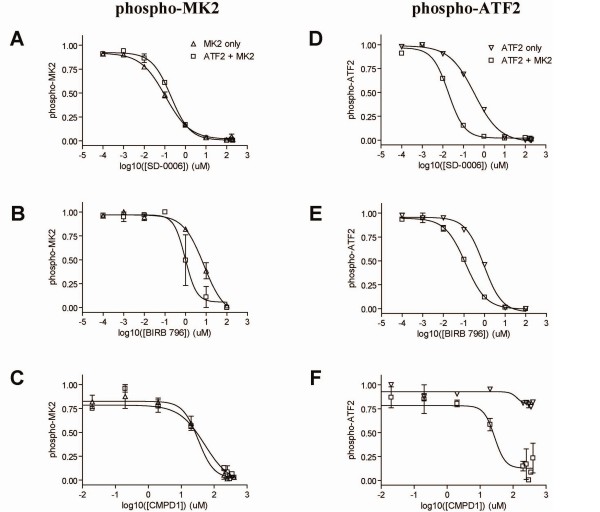
**Dual substrate assay with compounds**. Each compound was run in the dual substrate assay: *A*, SD-0006, *B*, BIRB 796, *C*, CMPD1, *D*, SD-0006, *E*, BIRB 796, *F *CMPD1. Phosphorylation of MK2 by p38 (left panels) was measured at 30 min for either MK2 alone (Δ) or MK2 + ATF2 (□). Phosphorylation of ATF2 by p38 (right panels) was measured at 120 min for either ATF2 alone (▽) or MK2 + ATF2 (□). Data is shown as a fraction of the maximum value ± standard deviation (n = 2) and IC_50 _values for each compound/substrate are listed in Table 3.

We next tested our set of compounds in the dual substrate assay. All compounds were run in the single and dual substrate assays and IC_50_'s determined for phospho-ATF2 and phospho-MK2 (Figure 11 & Table 3). As predicted by the model, there was no discernable effect on MK2 IC_50_. Further, the model correctly predicted a left-shift in ATF2 IC_50_, regardless of whether or not the compound was 'substrate-selective'. There were two novel findings that were correctly predicted via the modeling effort: (i) the substrate-selective inhibitor showed a loss of substrate-selective behavior in the dual substrate assays and (ii) the classical p38 inhibitors also showed a decrease in the ATF2 IC_50 _in the dual substrate assay. The loss of substrate-selectivity in the dual substrate assay was also replicated with a proprietary substrate-selective compound (data not shown).

**Table 3:** Dual Substrate Assay: Compound IC_50 _Values (uM)

*Compound*	*phospho-MK2*	*phospho-ATF2*	*substrate selectivity*
SD-0006	0.20 ± 1.1	0.02 ± 1.1	0.09
BIRB 796	3.6 ± 1.4	0.12 ± 1.1	0.03
CMPD1	41 ± 2.2	27 ± 16	0.66
Virtual Cmpd	0.0019 ± 1.1	0.0006 ± 1.2	0.33

IC_50_'s are shown ± standard error. Substrate Selectivity = (ATF2 IC_50_)/(MK2 IC_50_)

The modest left-shift predicted and experimentally observed in phospho-ATF2 IC_50 _in the classical inhibitors is due to the effect of MK2 on p38's ability to phosphorylate ATF2. The greater left-shift seen in substrate-selective inhibitors can be broken down into two parts: (i) the MK2-mediated effect on p38 and a second effect (ii): Mechanistically, a substrate-selective compound is designed to stabilize the p38-MK2 complex (in an inactive state). When compound is added, mass-action drives the formation of the p38-MK2-compound complex. Since [MK2] > [p38] in our assay design, all of the active p38 will be sequestered into the p38-MK2-inhibitor complex, reducing the pool of active p38 that is free to phosphorylate ATF2, in spite of the apparent 'substrate-selective' behavior seen in the single assays.

### Cell Characterization

**Figure 12:**
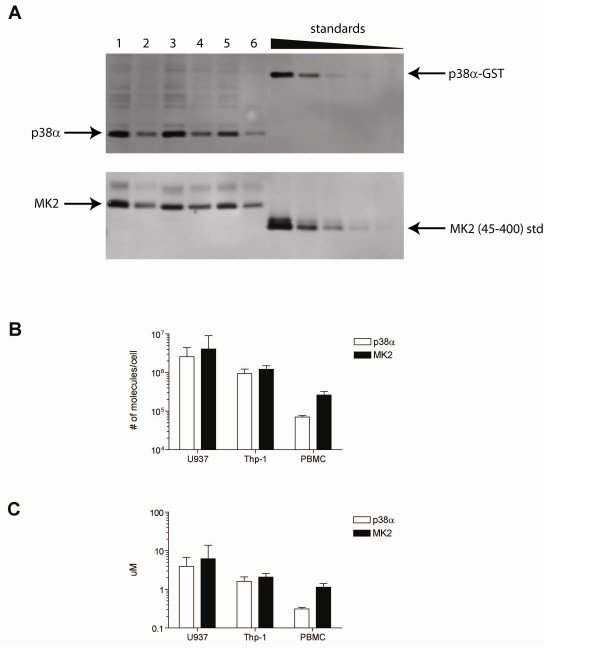
**Protein expression levels**. *A*, sample Western blots used for quantitation of total p38α and MK2 protein levels. On these representative blots, U937 cell lysates from 3 different experiments (lanes [1-4] and [5,6]) are loaded at 5 ug total protein (lanes 1, 3, 5) and 2 ug total protein (lanes 2, 4, 6), as measured by BCA assay. Recombinant p38-alpha and MK2 standards were loaded using 3-fold dilutions starting from 1.68 × 10^10 ^and 1.34 × 10^11 ^molecules per lanes, respectively. Cell lysates from U937 (n = 11, 13, for p38 and MK2 blots, respectively), Thp-1 (n = 4, 3) and PBMCs (n = 3, 4) were measured. Images were quantified with densitometry and total p38α (open bars) and MK2 (filled bars) protein expression levels were calculated. Results are shown in *B*, # of molecules/cell and in *C*, uM. Data is shown ± standard deviation.

Thus far, we have demonstrated both *in silico *and biochemically, that the presence of additional substrates results in the loss of substrate selectivity. Due to the sequestration effect of substrate selective compounds, we have found that a necessary condition for this to take place *in vivo *is that [active p38] < [MK2]. In order to see if this holds in relevant cell types, we measured protein expression levels of p38 and MK2 in the PMA-activated U937 and Thp-1 monocytic cell lines as well as in primary peripheral blood mononuclear cells (PBMCs). Protein expression was measured via Western blot on a per cell basis using recombinant standards and quantifying bands via densitometry, shown in Figure 12. In both U937 and Thp-1 cells, there are comparable protein levels of p38 and MK2 and in PBMCs the total protein level of MK2 significantly exceeds the total protein level of p38. Further, given that not all p38 in a cell is in the active conformation, our data strongly suggests that *in situ *[active p38] < [MK2]. This is also consistent with other reports of protein expression levels seen in MAPK signaling cascades [22]. Under these conditions one would expect the sequestration effect of substrate-selective compounds to take place.

## Discussion

The concept of a 'substrate-selective' inhibitor as a means to avoid unwanted side-effects is a very attractive one. The use of multiple screening assays to identify such compounds is a convenient and efficient method for identifying chemical entities with specific effects. However, great care should be taken to understand the cellular target(s) to determine the feasibility of such strategies particularly in more complex environments. From this work we specifically sought to explore the parameters governing the effectiveness of the substrate-selective inhibitor strategy in general and in particular for the p38-MK2 system.

On the path to understanding the behavior of substrate-selective inhibitors, an additional mechanism was uncovered: following interaction with MK2, the activity of p38 with regard to ATF2 is substantially reduced. From our analysis, there are multiple mechanisms that could give rise to this, including alteration of the affinity for ATF2 or the catalytic rate constant. Further determination of kinetic mechanism and molecular details was beyond the scope of this work. One might hypothesize that MK2 may, in some way, be eliciting an inhibitory phosphorylation on p38, however, this remains to be demonstrated.

Given that MK2 already has a much higher affinity for p38 than ATF2, one may ask how ATF2 would get phosphorylated at all within the cell (knowing that there is abundant cell-based experimental evidence to this end). In this case, one must recall that these are competing kinetic processes, rather than static events. Our dual substrate assay time course (Figure 2B) confirms that ATF2 phosphorylation continues at a measurable pace, albeit on a slower time-scale than MK2 (and including MK2's inhibition of p38-mediated ATF2 phosphorylation). Thus, abundant MK2 does not prevent p38 from phosphorylating ATF2 or other substrates, but merely slows it down. Simulations (Figure 6) further demonstrate that marked ATF2 phosphorylation is also quantitatively consistent with reported affinities of p38 for ATF2 and MK2. Even though p38 has markedly different affinities for ATF2 and MK2, we have demonstrated experimentally and computationally, that both substrates may get phosphorylated in a biochemical system with the key difference being the time scale over which they occur. Further, this work demonstrates that when ATF2 and MK2 are both present, a so-called 'p38 substrate-selective' inhibitor will inhibit the p38-mediated phosphorylation of both substrates comparably as a consequence of a sequestration phenomenon driven by an excess of MK2 relative to active p38.

We have used our computational model to predict that the introduction of multiple substrates would result in the loss of substrate selectivity and experimentally validated this finding in a biochemical assay. Alternately stated, the addition of MK2 to the p38-ATF2 reaction was able to make CMPD1 a potent inhibitor of ATF2 phosphorylation. Through the construction of a kinetic model of the proposed mechanism of action we demonstrate that these findings are a general result and not a compound-specific finding. Our analysis demonstrated that relative p38 and MK2 levels play a defining role in determining that the substrate-selective mechanism is not likely to work as intended *in vivo*. Additionally, this mechanism of sequestration-mediated inhibition of secondary substrates would extend to other substrates than ATF2 as well. It is worth noting that the presence of scaffolding proteins and higher-order interactions taking place in the cell that may locally alter protein concentration and drive interaction that would otherwise not take place in free solution. One cannot explicitly model such effects, however, given that their purpose is to locally increase protein concentration it is unlikely to change the outcome of our analysis.

In our kinetic model, we modeled each phosphorylation event as a one-hit reaction, even though p38 is known to phosphorylate MK2 and ATF2 at multiple sites (Thr 222, Ser 272 and Thr 334 for MK2 and Thr 69 and Thr 71 for ATF2). This was done to restrict the number of kinetic parameters in the model to as few as needed to adequately recapture the overall system behavior and be consistent with the experimentally measurable quantities, namely single phosphorylation sites.

Conceptually, one could consider p38-mediated phosphorylation or activation as the process by which p38 comes into contact with one of its substrates and performs the necessary modifications, either via one single-step reaction, or multiple dissociation and rebinding events. In order to perturb this process a compound needs to interfere with only one of these events. Our experimental data confirm that our compounds are able to interfere with MK2 and/or ATF2 phosphorylation, even though only single site was measured. If a substrate-selective inhibitor stabilizes the p38-MK2 interaction (or one of many possible forms) it will effectively sequester and prevent p38 from performing ATF2 phosphorylation, regardless of the number of phosphorylation sites. Consequently, the single phosphorylation site kinetic model was sufficient to describe and predict the observed behavior of both classical and substrate-selective model.

To further address the question of how multiple phosphorylation sites may affect a substrate-selective inhibitor we constructed a simplified model incorporating varying numbers of ATF2 and MK2 phosphorylation sites (Additional File 1 Supplementary Figure S1). It was found that increasing the number of ATF2 phosphorylation sites resulted in an increased potency against maximal ATF2 phosphorylation and this was independent of the number of MK2 phosphorylation sites. Similarly, increasing MK2 phosphorylation sites resulted in an increased potency against maximal MK2 phosphorylation (Additional File 2 Supplementary Figure S2). Importantly, these effects were independent of compound type. Thus, by having more MK2 phosphorylation sites than ATF2 phosphorylation sites, cells may naturally achieve substrate-selectivity. It is unclear what functional role, if any, this plays. Importantly, the incorporation of multiple phosphorylation sites did not affect the conclusion that under the condition where [MK2] > [active p38], a 'substrate-selective' inhibitor will inhibit the phosphorylation of both MK2 and ATF2.

In three monocytic cell systems we have shown that total MK2 protein expression does not exceed that of p38 in resting cells. In a resting cell inactive MK2 is reported to reside in the nucleus and the active form of p38 undergoes nuclear import [23]. Thus, when considering this compartmentalization, the level of MK2 may even further exceed the level of active p38. Consequently, it is unlikely that substrate selectivity can be achieved for any nuclear substrates of p38. However, due to the compartmentalization and the nuclear/cytoplasmic shuttling of the active forms, it may be feasible to obtain substrate-selectivity for cytosolic substrates. In these cases, however, substrate selectivity is not a general property and will vary based on substrate levels and substrate-dependent kinetic interaction rates.

Our analysis has centered on a simplified model and experimental system and may differ from *in vivo *situations in a number of ways. For example, this work did not take into account the mechanism by which p38 gets activated, whether it be via upstream kinases or via autophosphorylation [24]. It is possible that various inhibitors may bind differently to active and inactive forms of p38. Further, many p38 compounds have been observed to inhibit p38 activation as well [25]. Neither of these phenomena would be expected to affect the stoichiometric effect by which MK2 levels would soak up all available active p38 when treated with 'substrate-selective' inhibitors. When one considers that there are multiple converging pathways on ATF2, MK2 (and other substrates), as well positive and negative feedback loops that regulate activity and expression levels of enzymes and substrates, accurately predicting overall behavior becomes considerably more complex. Nonetheless, our kinetic model provides initial guidance for how a sub-system of the signaling network may operate and serves as a building block for adding in additional complexity.

## Conclusions

The analysis presented herein suggests that the substrate-selective mechanism of inhibiting is not an effective strategy for p38/MK2/ATF2 system in cases where the concentration of MK2 exceeds the concentration of p38. This work may point to general properties regarding the substrate-selective inhibitor concept. With regard to other secondary substrates, beyond ATF2, we believe they will also be inhibited due to the stoichiometric excess of MK2 relative to active p38. Further, we believe this work contributes to determining biological conditions that are required for the substrate-selective inhibitor strategy to be effective. There may be other kinase-substrate pairs in which the stoichiometry is not limiting for the substrate-selective mechanism and our computational model is easily adapted for their evaluation. However, moving closer to the receptor level may broaden the downstream effects by virtue of being farther from transcriptional and translational endpoints. Overall, targeting kinase-substrate complexes may offer a general approach and new avenue for achieving selectivity for kinases with high structural similarity to other proteins. The different requirements of targeting the p38-MK2 complex vs. free p38 may lead us into new chemical space that could still find alternate avenues to differentiate from previous p38 inhibitors.

## Methods

### Biochemical Assay

The ability of active p38 to phosphorylate ATF2, MK2 or both was tested in a biochemical assay. Reagents were added in the following order: compound, substrate(s), ATP, p38 for a final reaction volume of 40 ul in low protein binding 96-well plates (Immulon-1B). Final reaction conditions were 0.5-5 nM p38 (in-house, MKK6-activated), 50 uM, 100 nM ATF2 (Santa Cruz Biotechnology SC-4007) 10 nM MK2 (Upstate 14-349). Reactions were performed in buffer containing 20 mM HEPES pH 7.5, 10 mM MgCl_2_, 0.01% BSA, 0.0005% Tween-20, 2% DMSO and 0.1 mM DTT. Reactions were quenched with 40 ul of buffer containing 50 mM HEPES pH 7.5, 30 mM EDTA following different incubation times. Phosphorylated ATF2 and phosphorylated MK2 were assayed as described below. Compounds were diluted in DMSO (Sigma).

### Phospho-ATF2 Assay

Phospho-ATF2 was measured with the Meso-Scale Discovery (MSD) platform using an in-house assay. High-binding MSD plates were spotted with 5 ul of 25 ug/ml rabbit anti-ATF2 (Santa Cruz Biotechnology SC-187, 25 ug/ml in TBS) and left to dry overnight. Plates were blocked with 3% MSD Blocker A in MSD Tris Wash Buffer for at least 1 hr at RT. 25 ul of sample was added with 25 ul of antibody cocktail containing 0.2 ug/ml mouse anti-phospho ATF2 (Santa Cruz Biotechnology SC-8398) and 1 ug/ml goat anti-mouse sulfo-tag (MSD R32AC-1) diluted in 1% Blocker A in Tris Wash. Plates were incubated for > 3 hr at RT, washed 4× with 150 ul MSD Tris Wash Buffer and read on the Meso-Scale Discovery Sector imager using 2× MSD Read Buffer T.

### Phospho-MK2 Assay

Phospho-MK2 was measured with the MSD platform using an in-house assay. High-binding MSD plates were spotted with 5 ul of 25 ug/ml goat anti-MK2 (SC-6221, 25 ug/ml in TBS) and left to dry overnight. Plates were blocked with 3% MSD Blocker A in MSD Tris Wash Buffer for at least 1 hr at RT. 25 ul of sample was added with 25 ul of antibody cocktail containing 1 ug/ml mouse anti-phospho MK3 (Thr 334, in-house antibody) and 1 ug/ml goat anti-rabbit sulfo-tag (MSD R32AB-1) diluted in 1% Blocker A in Tris Wash Buffer. Plates were incubated for > 3 hr at RT, washed 4× with 150 ul MSD Tris Wash Buffer and read on the Meso-Scale Discovery Sector imager using 2× MSD Read Buffer T.

### Immunoprecipitation Experiment

Anti-total p38 (A12, Santa Cruz Biotechnology SC-7972) at 1 ug/ml was bound to Immobilized Protein G (Pierce 20398). 5 nM p38 was incubated with varying amount of MK2 for 1 hour at RT and then added to p38 antibody bound to protein G and incubated o/n at 4°C. The next day antibody and protein G were washed once with enzyme buffer, once with IP Buffer (1× TBS, 500 mM NACl, 20 mM Tris, pH 7.5) and twice more with enzyme buffer ending in fresh enzyme buffer for the second kinase reaction with ATF2. After 1 hour stop buffer was added and assayed for phospho-ATF2 in the MSD assay.

### Cell Culture

U937 and Thp-1 cells were obtained from ATCC and cultured under the recommended conditions. Prior to cell plating, cells were differentiated into macrophages with PMA (Sigma). Peripheral blood mononuclear cells (PBMCs) were isolated from whole blood using Ficoll gradient centrifugation and maintained in RMPI + 10% FBS.

### Quantitative Western Blotting

U937, Thp-1 cells and PBMCs were counted and lysed. Following total protein determination via BCA assay (Pierce), cells/ug was calculated. Cell lysates were run on SDS-PAGE gels (Invitrogen) at 2 and 5 ug total protein per well alongside a 5 point standard of recombinant protein (p38-alpha (in-house, full length-GST tagged), MK2 (in-house, residues 45-400)). Three-fold dilutions of standards were loaded, starting at 1.68 × 10^10 ^and 1.34 × 10^11 ^molecules per well for p38-alpha and MK2, respectively. p38-alpha blots were probed with rabbit anti-p38alpha (Cell Signaling Technology #9218). MK2 blots were probed with rabbit anti-MK2 (Cell Signaling Technology #3042). Blots were imaged and bands quantified using densitometry. Protein standards were used to construct a standard curve, from which the number of molecules per cell could be calculated. Numbers were converted into uM, based on reported cell volumes for the different cell types: 1.09 pl, 0.97 pl and 0.38 pl for U937, Thp-1 and PBMC, respectively [26-28].

### Kinetic Model Development

A mass-action kinetic model was built to precisely simulate the experimental biochemical reaction conditions. In the kinetic model, activated p38 reversibly binds ATP with affinity *K*_D, ATP_, independent of further complex formation. p38 (ATP bound or not) may reversibly complex with either ATF2 or MK2 yielding p38-ATF2, p38:ATP:ATF2, or p38:MK2, p38:ATP:MK2. Complex formation is characterized by affinities *K*_D, ATF2 _and *K*_D, MK2_. p38:ATP:ATF2 and p38:ATP:MK2 undergo a irreversible catalysis step yielding phospho-ATF2 and ADP with rate constant *k*_cat, ATF2 _or phospho-MK2 and ADP with rate constant *k*_cat, MK2_. It is known that p38 phosphorylates ATF2 on Thr69 and Thr71 in a two-step distributive mechanism [19,29] and MK2 on multiple sites [13], however, for the sake of simplicity we have modeled phosphorylation as a one-step process. Rate constants and literature references are listed in Table 2. All forward interaction rates are assumed to be diffusion limited (ATF2 and MK2 forward interaction rate constants with p38 were set to 10^6 ^M^-1^s^-1^; ATP and compound forward interaction rate constants were set to 10^7 ^M^-1^s^-1^). Simulations were performed in a 40 ul reaction volume, with 0.5 nM p38, 100 nM ATF2, 10 nM MK2 and 50 uM ATP.

### Additional Kinetic Mechanisms

[1] Substrate Inhibition: phospho-MK2 and phospho-ATF2 are allowed to re-bind p38 to form phospho-MK2:p38 and phospho-ATF2:p38. Interactions are assumed to occur at rates equal to the unphosphorylated interactions. [2] phospho-MK2 binds ATF2: phospho-MK2 is allowed to bind unphosphorylated ATF2 to form phospho-MK2:ATF2 and thereby block its interaction with and phosphorylation by p38. It is assumed that this interaction occurs with the same affinity as the p38-ATF2 interaction [3-5]. After interaction with MK2, p38 is left in a modified form, p38*. p38* undergoes the same interactions as before with the same kinetic constants except for: [3] K_D, ATP _is lowered 10-fold; [4] K_D, ATF2 _is lowered 10-fold; and [5] k_cat, ATF2 _is lowered 10-fold.

### Computations

The kinetic model was coded in Teranode Design Suite (Teranode Corporation, Seattle, WA). The completed model was exported in SBML format http://www.sbml.org. The SBML code was translated into the Jacobian language (Numerica Technology, Cambridge, MA) using a translator written in MATLAB (The Mathworks, Natick, MA) using the SBML toolbox http://sbml.org/software/sbmltoolbox/. Scripts were written in Jacobian and MATLAB to generate dose-response curves and figures. Model files are available in Additional Files 3, 4, 5 and 6. IC_50_'s were calculated using GraphPad Prism (GraphPad Software, La Jolla, CA).

## Abbreviations

RA: rheumatoid arthritis; LPS: lipopolysaccharide; TNF: tumor necrosis factor alpha; ATF2: activating transcription factor 2; MK2: mitogen-activated protein kinase-activated protein kinase 2; p38: p38 MAP kinase; IC_50_: the concentration of an inhibitor that is required for 50% inhibition of an enzyme in vitro; MSD: Meso-Scale Discovery.

## Competing interests statement

BSH is currently employed by Merrimack Pharmaceuticals. KMS is currently employed by Novartis Pharmaceuticals. JRC is currently employed by Pfizer, Inc. All authors hold stock in Pfizer, Inc.

## Financial competing interests

All authors hold stock in Pfizer, Inc.

## Authors' contributions

All authors contributed to the design of experiments. BSH and JRC constructed and analyzed the kinetic models. BSH and KMS performed the experiments. BSH drafted the manuscript. All authors read and approved the final manuscript.

## Supplementary Material

Additional file 1 Supplementary Figure S1 Click here for file

Additional file 2 Supplementary Figure S2 Click here for file

Additional file 3 **SSImodel**. Substrate-selective inhibitor model in SBML (systems biology markup language http://www.sbml.org) format. All model variations described in the text are included. Click here for file

Additional file 4 **SSImodel**. Substrate-selective inhibitor model translated into format for Jacobian modeling software (http://www.numericatechnology.com, free for academia). This file includes scripts used to generate simulated data for dose-response curves. Click here for file

Additional file 5 **SSImodel_expanded**. Supplementary model - expanded substrate-selective inhibitor model including multiple phosphorylation sites (used for supplementary figures). This model is in MATLAB SimBiology (R2009a) format. Click here for file

Additional file 6 **SSImodel_expanded**. Supplementary model, exported into SBML (systems biology markup language http://www.sbml.org) format. Click here for file
