# Supplementary figures and images for: Two additive mechanisms impair the differentiation of 'substrate-selective' p38 inhibitors from classical p38 inhibitors in vitro
Source: BMC Syst Biol. 2010 Mar 15;4:23. doi: 10.1186/1752-0509-4-23 (PMC2850882; doi:10.1186/1752-0509-4-23)

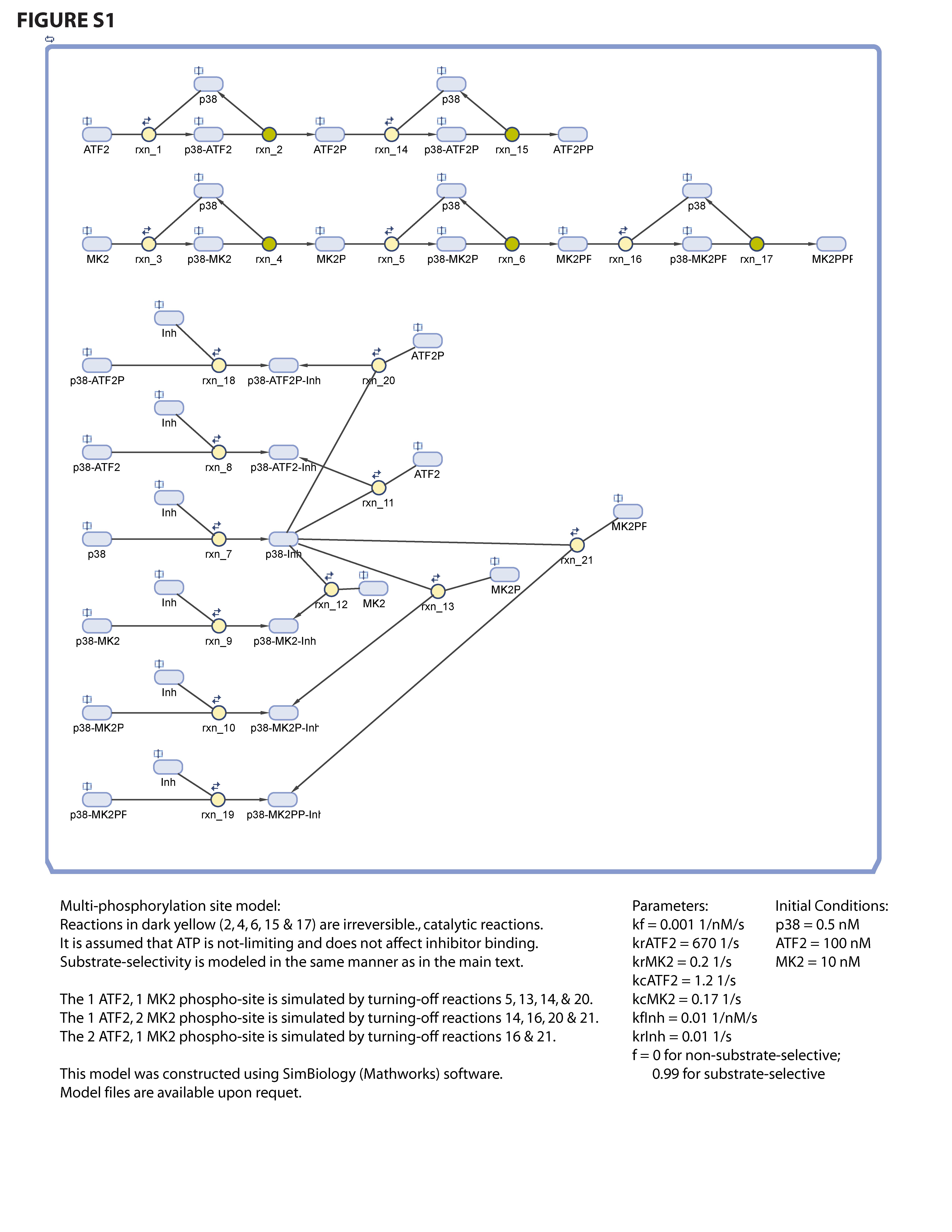

Supplement: Additional file 1 — Supplementary Figure S1 [file 1752-0509-4-23-S1.JPEG]

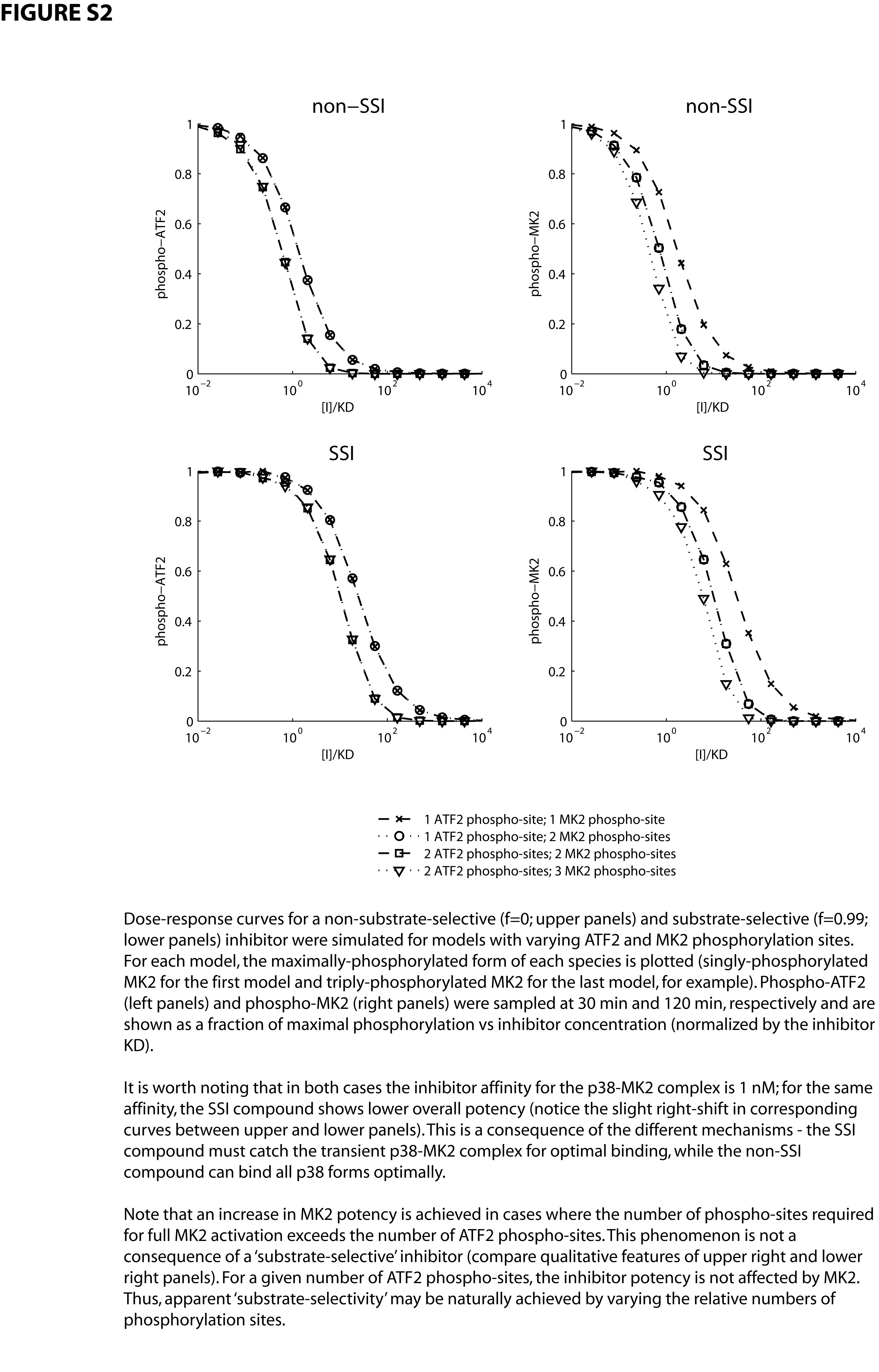

Supplement: Additional file 2 — Supplementary Figure S2 [file 1752-0509-4-23-S2.JPEG]
